# Efficacy and safety of traditional Chinese medicine for erosive oral lichen planus: A protocol for systematic review and meta analysis

**DOI:** 10.1097/MD.0000000000023375

**Published:** 2020-11-25

**Authors:** Yanyan You, Xiaojin Huang, Yunhui Chen, Yu You

**Affiliations:** a West China Hospital of stomatology, Sichuan University, South Renmin Road, Wu Hou District; b Chengdu University of Traditional Chinese Medicine, Chengdu, China.

**Keywords:** Erosive oral lichen planus, Meta-analysis, Protocol, Randomized controlled trials., systematic review, Traditional Chinese Medicine

## Abstract

**Background:**

Oral lichen planus (OLP) is a common disease among oral mucous membrane diseases. Erosive oral lichen planus (EOLP) is a type of OLP, it has a potential tendency of cancerization. There have been some randomized controlled trials (RCTs) using Traditional Chinese Medicine (TCM) to treat EOLP. No systematic review on the RCTs of TCM for EOLP has been reported, so we would propose a study protocol that aims to evaluate the evidence the efficacy and safety of TCM for treating patients with EOLP.

**Methods:**

The following databases from the inception to June 30, 2020 electronically, including PubMed, EMBASE, Cochrane Library, Chinese National Knowledge Infrastructure, VIP, Wanfang database, China Biomedical Literature Database will be searched. RCTs that meet the pre-specified eligibility criteria will be included. RevMan software (V5.3) will be performed data synthesis following data extraction and publication risk assessment. Subgroup and sensitivity analysis will be performed according to the condition of included RCTs. The primary outcomes include visual analogy scale, laboratory immune indicators, and scores of oral lesions and signs. Additional outcomes are clinical effective rate, adverse event rate, and recurrence rate. The Grading of Recommendations Assessment, Development and Evaluation system will be used to assess the strength of the evidence.

**Results:**

This study will provide a well-reported synthesis of RCTs on the efficacy and safety of TCM in the treatment of EOLP.

**Conclusion:**

This systematic review protocol will be helpful for providing evidence of whether TCM is an effective and safe therapeutic approach for patients with EOLP.

**Ethics and dissemination:**

Ethical approval is not necessary as this protocol is only for systematic review and it does not involve privacy data or conduct an animal experiment. This protocol will be disseminated by a peer-review journal or conference presentation.

**Systematic review registration:**

PROSPERO CRD42020172366.

## 1 Introduction

Oral lichen planus (OLP) is a chronic oral mucosal inflammatory disease of unknown etiology, which is the most common type of oral mucosal diseases excepting recurrent aphthous ulcer,^[1]^ and the prevalence of it is 0.2% to 2.3%.^[2]^ The local lesions of the oral cavity are gray white keratosis papules with white stripes, which can be divided into reticular type, dendritic stripe type, annular type, plaque type, blister type, erosion type, papule type, and atrophic type according to the morphology characteristics, in fact, some patients were found to have pain by chance or in case of spicy stimulation. Immunosuppressor and glucocorticoid are used for the treatment of OLP in western medicine (WM),^[1,3]^ but long-term application of WM causes some problems like more adverse reactions, weakened immunity, and easy relapse after drug withdrawal. Traditional Chinese medicine (TCM) regulates function of viscera to treat diseases based on the holistic concept and syndrome differentiation, which can better avoid the above problems.^[4–5]^ World Health Organization (WHO) has classified this disease into the category of precancerous conditions,^[6–7]^ in addition, prep-study has shown that the carcinogenesis rate of erosive oral lichen planus (EOLP) is 0.4% to 12.3%,^[8]^ so more attention should be paid to it. At present, there have been some randomized controlled trials (RCTs) using TCM to treat EOLP. To the best of our knowledge, no systematic review on the RCTs of TCM for EOLP has been reported, so we would propose a protocol for a systematic review to evaluate the evidence of TCM's efficacy and safety for treating patients with EOLP.

## 2 Methods

### 2.1 Study registration

This systematic review protocol has been registered on PROSPERO (www.crd.york.ac.uk/prospero/) with number CRD42020172366. Ethical approval is unnecessary because this study only involves the data of previous studies.

### 2.2 Eligibility criteria

#### 2.2.1 Type of study

Only RCTs can be included. Observation studies, animal research, case report, review, and meta-analysis are excluded.

#### 2.2.2 Participants

Patients with EOLP were diagnosed by biopsy or clinical manifestations according to the < Oral Medicine > . There is no restriction on age, gender, race and the duration and severity of the disease.

#### 2.2.3 Interventions

EOLP patients treated with TCM with or without combing conventional WM treatment. TCM in the study is defined as herbal formula. The dosage form of TCM mainly include decoction, granule.

#### 2.2.4 Comparison

EOLP patients treated with the conventional WM regimen were chosen as intervention group in the original study. There no restriction on regarding conventional WM treatment regimen.

2.2.5 Outcome. The primary outcomes include visual analogy scale, laboratory immune indicators, scores of oral lesions and signs. Additional outcomes are clinical effective rate, adverse events, and recurrence rate.

#### 2.2.5 Language

There is restriction on Chinese or English

### 2.3 Information source

We will search the following databases from the inception to June 30,2020 electronically, including PubMed, EMBASE, Cochrane Library, Chinese National Knowledge Infrastructure, VIP, Wanfang database, China Biomedical Literature Database.

### 2.4 Search strategy

The electronic search will be conducted using a combination of following keywords

OLP, EOLP, TCM, Chinese Herbal Medicine, Chinese and WM, RCT, Manual searches will also be conducted to identify the extra studies from the reference list. The search strategy for PubMed is presented in Table 1 and strategy will be modified upon the requirement of other databases.

**Table 1:** Search strategy for the PubMed.

NO.	Search terms
#1	Erosive oral lichen planus
#2	Traditional Chinese Medicine
#3	Chung I Hsueh
#4	Hsueh, Chung I
#5	Traditional Medicine, Chinese
#6	Zhong Yi Xue
#7	Chinese Traditional Medicine
#8	Chinese Medicine, Traditional
#9	Traditional Tongue∗
#10	Tongue Diagnoses, Traditional
#11	Tongue Assessment, Traditional
#12	#2 OR #3 OR #4 OR #5 OR #6 OR #7 OR #8 OR #9 OR #10 OR #11
#13	Randomized controlled trial
#14	Controlled clinical trial
#15	randomized
#16	randomly
#17	trials
#18	RCT
#19	#13 OR #14 OR #15 OR #16 OR #17 OR #18
#20	#1 AND #12 AND #19

### 2.5 Data collection and analysis

#### 2.5.1 Study selection

**Figure 1:**
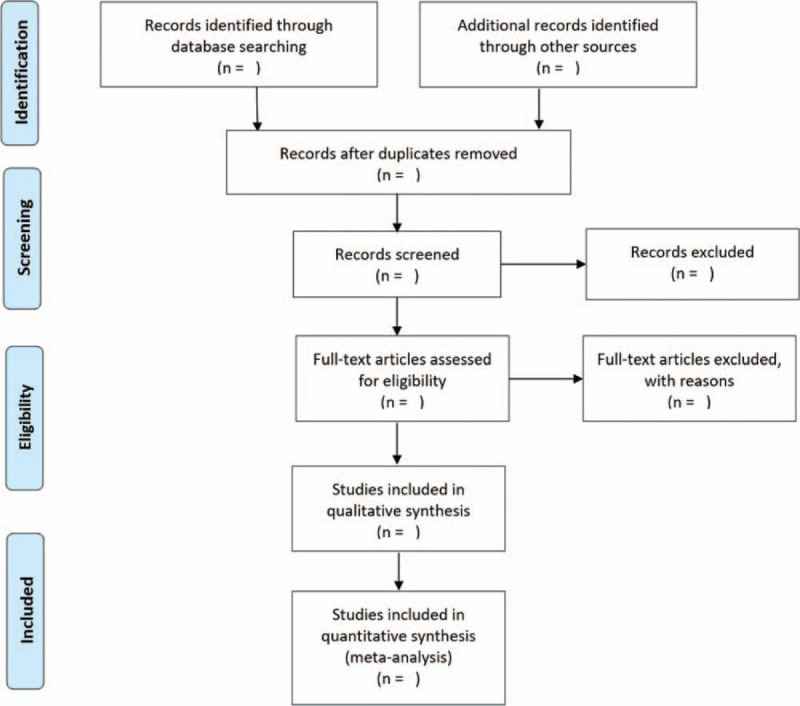
Flow chart of study selection.

Two reviewers will perform literature screening, study selection and data extraction independently. The literature obtained will be imported into NoteExpress to screen the title and abstract, and the duplications and studies failing to meet the pre-specified inclusion criteria will be excluded. After reading the full text of the remaining studies, the final included studies will be determined. The corresponding author from an original study will be contacted when the full text is unavailable. Any disagreements will be arbitrated by a third reviewer. The entire process of study selection is presented in a PRISMA flow chart (Fig. 1)

#### 2.5.2 Data extraction and management

Two reviewers will independently extract data with a pre-specified standard form, including general information (title, serial number, first author, the country, publication name, and publication date), participants characteristics (age, gender, duration, and number), interventions of trial and control group (study design, sample size, details of randomization, blinding, allocation, intervention approach, Chinese medicine prescription and duration), primary outcomes, and additional outcomes. Inconsistency between two reviewers will be solved by a third reviewer.

#### 2.5.3 Risk of bias in included studies

Two reviewers will assess the risk of publication bias for every included RCT with the Cochrane Risk of Bias Tool independently in terms of sit items, including random sequence generation, allocation concealment, blinding of participants and researchers, incomplete outcome data, selective reporting bias, and other bias, each item will be grade high, unclear, or low risk of bias. Inconsistency will be solved by consultation with a third reviewer.

#### 2.5.4 Measurement of treatment effect

Two reviewers will analyze the data independently using RevMan 5.3. Risk ratio with 95% confidence interval will be adopted for the dichotomous data, whereas the mean difference or standardized mean difference with 95% confidence intervals will be utilized for the continuous data.

#### 2.5.5 Management of missing data

If required data is unclear, miss or difficult to be obtained reliably, the corresponding author of the original RCT will be contacted by e-mail or telephone. If data is still unattainable, the study concerned will be excluded from the data analysis.

#### 2.5.6 Assessment of reporting biases

A funnel plot will be performed to assess any publication bias when more than 10 RCTs are included. In addition, Egger regression and Begger correlation test will also be performed to identify the funnel plot asymmetry.

#### 2.5.7 Assessment of heterogeneity

The Cochrane *I*^*2*^ and *Χ*^*2*^ tests will be applied to evaluate the heterogeneity with the cut-off value of *I*^*2*^ = 50. Fixed-effects model will be applied when heterogeneity is low (*I*^*2*^ < 50%), and random-effects model will be used for moderate heterogeneity (50% < *I*^*2*^ < 75%). When heterogeneity is considerably high, meta-analysis will not be performed.

#### 2.5.8 Data synthesis

In line with the Cochrane guideline, the fixed-effect model will be utilized to pool and analyze the outcome data if heterogeneity is deemed low and the random-effect model will be employed if heterogeneity is deemed moderate. Subgroup analysis or meta-regression will be performed to assess the potential sources and present reasonable explanations for the heterogeneity. The statistical significance is defined as *P* < .05. If the meta-analysis is not feasible, a narrative description of the results will be provided.

#### 2.5.9 Subgroup analysis and investigation of heterogeneity

If feasible, subgroup analyses will be performed based on the preparations, dosage, ingredients of TCM interventions that included patients. Heterogeneity will be explained by subgroup analysis.

#### 2.5.10 Sensitivity analysis

If feasible, sensitivity analysis will be applied to evaluate the stability of the pooled results of include RCTs according to the methodological quality, sample size and missing data.

#### 2.5.11 Grading the quality of evidence

The Grading of Recommendations Assessment, Development and Evaluation guidelines will be utilized to grade the quality of evidence as very low, low, moderate, or high.

## 3 Discussion

Etiology of OLP is unknown, there is much evidence that OLP is associated with autoimmune function.^[9,10]^ Some OLP patients go undetected for a long time because they have no obvious symptoms, but for EOLP, oral pain can seriously affect the quality of life of patients. EOLP has a potential tendency of cancerization, which will increase the emotional burden of patients. Through syndrome differentiation and treatment, TCM adjusts of qi movement, balances Yin and Yang of the body and restores the normal physiological functions of the zang-fu organs (viscera). Modern pharmacology also proves that part of TCM has the function of immune regulation, anti-inflammation, promote metabolism,^[11–14]^ and improve microcirculation.^[15,16]^

However, currently no systematic review and meta-analysis have been conducted regarding the efficacy and safety of TCM for the treatment of patients with EOLP. This study systematically evaluated the safety and efficacy of TCM in the treatment of EOLP, in order to provide evidence-based medical evidence for treatment of EOLP with TCM.

## Author contributions

**Conceptualization:** Yanyan You, Xiaojin Huang.

**Formal analysis:** Yanyan You, Yunhui Chen, Yu You.

**Funding acquisition:** Yu You

**Investigation:** Yanyan You, Yunhui Chen.

**Methodology:** Yanyan You, Yunhui Chen, Xiaojin Huang.

**Validation:** Xiaojin Huang, Yu You.

**Writing – original draft:** Yanyan You, Xiaojin Huang

**Writing – review & editing:** Yunhui Chen, Yu You.
